# Thermostable Human Basic Fibroblast Growth Factor (TS-bFGF) Engineered with a Disulfide Bond Demonstrates Superior Culture Outcomes in Human Pluripotent Stem Cell

**DOI:** 10.3390/biology12060888

**Published:** 2023-06-20

**Authors:** Sejong Kim, Geun-Ho Kang, Kyung Min Lim, Yeokyung Shin, Kwonwoo Song, Sangrok Park, Jongyub An, Dae Young Kim, Hang-Cheol Shin, Ssang-Goo Cho

**Affiliations:** 1 Department of Stem Cell and Regenerative Biotechnology, Molecular & Cellular Reprogramming Center and Institute of Advanced Regenerative Science, Konkuk University, 120 Neungdong-ro Gwangjin-gu, Seoul 05029, Republic of Korea; rlatpwhdc@nate.com (S.K.); geunhokang@naver.com (G.-H.K.); lmin0217@naver.com (K.M.L.); cutiesally@naver.com (Y.S.); rnjsdnthd814@naver.com (K.S.); packsangrok@naver.com (S.P.); delrar@naver.com (J.A.); 2 R&D Team, StemExOne Co., Ltd., 307 KU Technology Innovation Bldg, 120, Neungdong-ro Gwangjin-gu, Seoul 05029, Republic of Korea; 3 PnP Biopharm Co., Ltd., 1304, Acetechnotower 8-cha, 11 Digital-ro 33-gil, Guro-gu, Seoul 08380, Republic of Korea; kimdae@pnpbiopharm.com

**Keywords:** growth factor, bFGF, human pluripotent stem cells, thermostable, TS-bFGF

## Abstract

**Simple Summary:**

Human pluripotent stem cells can differentiate into various tissues, making them an important source for cell therapy products. However, culturing pluripotent stem cells requires various growth factors, among which basic fibroblast growth factor is essential for maintaining stem cell ability. Unfortunately, basic fibroblast growth factor has a short half-life during cell culture and requires continuous supply, posing a significant challenge to culturing high-quality stem cells. To address this issue, we developed a thermostable basic fibroblast growth factor that is thermally stable and can maintain its activity for a longer period of time. We evaluated the various functions of human embryonic stem cells using thermostable basic fibroblast growth factor and found that cells cultured with thermostable basic fibroblast growth factor showed better proliferation, stemness, morphology, and differentiation. Thus, thermostable basic fibroblast growth factor with high heat resistance and persistence can play a critical role in securing high-quality stem cells during their cultivation.

**Abstract:**

Human embryonic stem cells (hESCs) and induced pluripotent stem cells (iPSCs) can differentiate into various tissues and are an essential source of various disease models and therapeutics. Various growth factors are required in order to culture pluripotent stem cells, among which basic fibroblast growth factor (bFGF) is essential for maintaining stem cell ability. However, bFGF has a short half-life (8 h) under normal mammalian cell culture conditions, and its activity decreases after 72 h, posing a serious problem in the production of high-quality stem cells. Here, we evaluated the various functions of pluripotent stem cells (PSCs) by utilizing an engineered thermostable bFGF (TS-bFGF) that is thermally stable and maintains activity longer under mammalian culture conditions. PSCs cultured with TS-bFGF showed better proliferation, stemness, morphology, and differentiation than cells cultured with wild-type bFGF. In light of the importance of stem cells in a wide range of applications in the medical and biotechnology fields, we anticipate that TS-bFGF, as a thermostable and long-acting bFGF, can play a key role in securing high-quality stem cells through various sets of stem cell culture processes.

## 1. Introduction

Human embryonic stem cells (hESCs) and induced pluripotent stem cells (iPSC) are pluripotent cells (PSCs) that give rise to all somatic cell types. PSCs are capable of differentiating into all tissues and organs of adult animals, including the ectoderm, mesoderm, and endoderm [1], under appropriate induction conditions [2]. PSCs can proliferate endlessly and sustain self-renewal under appropriate cell culture conditions [3]. PSCs have the ability to be maintained indefinitely in culture as undifferentiated cells, yet are able to form more differentiated cell types. The use of PSCs to replace damaged tissues in patients suffering from various degenerative diseases has been reported [4]. 

Indefinite self-renewal is a fundamental hallmark of successful PSC cultures. Various growth factors, including basic fibroblast growth factor (bFGF), transforming growth factor-β (TGF-β), epidermal growth factor (EGF), insulin-like growth factor (IGF), and platelet-derived growth factor (PDGF), are involved in maintaining pluripotency in PSCs [5,6,7,8]. Among the various growth factors, exogenous bFGF is required to sustain self-renewal of hESCs and their capacity to differentiate into a large number of somatic cell types. It is possible to grow PSCs in a serum-free culture medium containing bFGF in trophoblasts [9]. bFGFs can affect the proliferation of multiple cells, including endothelial cells, neurons, chondrocytes, smooth muscle cells, melanocytes, and adipocytes [10]. In addition, bFGF is involved in a variety of biological processes, including proliferation, self-renewal, differentiation, development, tissue repair, and regeneration in PSCs. The maintenance of PSCs requires the activation of activin and FGF signaling [11]. Therefore, bFGF was initially used to maintain PSCs in an undifferentiated state under feeder-free conditions [12,13]. Moreover, FGF signaling pathways have been found to play critical roles in modulating PSCs by inhibiting differentiation [14]. The results of these studies indicate that bFGF, as a key growth factor, is essential for maintaining the pluripotency of PSCs.

bFGF has been shown to be unstable at physiological conditions, with a half-life of approximately 8 h in mammalian cell culture conditions. Therefore, application of commercialized bFGF is limited due to its rapid loss during cultivation [15,16]. The instability of bFGF causes problems with cell growth, often requiring supplementation of growth factors during cell culture or in certain cases by changing the medium daily. Therefore, there is an urgent need to develop bFGF with excellent stability in order to maintain the cell characteristics during PSC culture. 

Based on the complex structure of bFGF and its receptor [17], we analyzed the critical residues of bFGF for receptor binding and stability, then generated bFGF variants by introducing mutations, including a disulfide linkage. After thorough characterization, TS-bFGF, a thermostable and long-acting bFGF, was selected due to its superiority over WT-bFGF in cellular regeneration processes such as wound healing [18]. Based on the critical role of bFGF in stem cell culture, in this study we used TS-bFGF to evaluate the characteristics of PSCs compared to their WT counterparts, demonstrating that PSCs cultured with TS-bFGF have better characteristics in terms of cell proliferation, stemness, morphology, and cell differentiation. We anticipate that TS-bFGF can be applied to various cell culture processes to produce high-quality stem cells.

## 2. Materials and Methods

### 2.1. Computational Design of Thermostable-bFGF (TS-bFGF)

Amino acid residues involved in receptor binding, heparin binding, and conforma-tional stability were analyzed based on the complex structure of bFGF and its receptor [17]. Excluding residues directly involved in receptor binding and heparin binding, we selected fifteen candidate residues that may influence protein stability and constructed mutant structures using the Swiss Model [19]. Energy minimization and free energy change of mutation (ΔΔG) were calculated using YASARA [20] and FoldX [21].

### 2.2. TS-bFGF Construction and Production

The TS-bFGF gene cloned into our in-house bacterial expression vector was expressed in BL21 codon+ (DE3) cells (Novagen). TS-bFGF was expressed as a soluble protein using a high-cell density culture in a 10 L scale jar fermenter according to our own protocol. In brief, a seed culture prepared prior to main culture was added into 6 L of main culture media and cultured at 37 °C until pH of the culture reached about 7.1. Then, media for feeding was added and cultured until OD600nm reached 60 when gene expression is induced with IPTG (Isopropyl β-D-1-thiogalacto pyranoside) and temperature is set from 37 °C to 25 °C. Another IPTG was added for the second induction at 4 hours after the first induction, and the culture was finished at 4 hours after the second induction. Over the course of the culture, the bacterial cultures were sampled every hour and used to monitor the expression of TS-bFGF using SDS-PAGE (Sodium dodecyl sulfate–polyacrylamide gel electrophoresis). The cultured bacterial cells were harvested by centrifugation and the cells stored at −20 °C until needed for purification. The soluble form of TS-bFGF was applied to an S-Sepharose column equilibrated with 20 mM sodium phosphate buffer (pH 7.0) eluted with 20 mM sodium phosphate buffer (pH 7.0) containing 1 M NaCl and subsequently subjected to heparin-Sepharose column chromatography for further purification. Purified TS-bFGF was oxidized by treatment with different amounts of cysteine and cystine under alkaline pH conditions, followed by ultrafiltration for buffer change and concentration. Lastly, purified TS-bFGF was stored as a lyophilized powder at −20 °C.

### 2.3. Determination of Thermal Stability

To determine thermal stability of WT-bFGF and TS-bFGF, the secondary structures of the two bFGFs were analyzed using a spectropolarimeter (J-810; JASCO, Tokyo, Japan). Far-ultraviolet circular dichroism (CD) spectra (190 nm–250 nm, 25 °C, 1-mm path length) and temperature scans (20 °C–95 °C at 220 nm, 0.1-mm path length) were evaluated by monitoring the change in CD at 200 nm. The temperature of the protein solution (0.2 mg/mL) in 20 mM sodium phosphate buffer (pH 7.0) was increased in 1 °C increments from 20 °C to 95 °C [22]. Thermal denaturation curves were calculated as previously described [23].

### 2.4. Cell Proliferation Assay Using NIH/3T3 Cell Line

To assess cell proliferation activity of WT-bFGF and TS-bFGF, both of which were stored in solution (0.1 mg/mL in 20 mM sodium phosphate buffer, pH 7.0, for both) at 25 °C for a period of 12 months and sampled every determined interval for assay, a cell viability assay was performed using the NIH/3T3 fibroblast cell line (ATCC). Cells were seeded at a density of 5 × 10^3^ cells/well in 96-well plates and cultured for 24 h in high-glucose Dulbecco’s modified Eagle’s medium (DMEM) supplemented with 10% fetal bovine serum and 1% penicillin/streptomycin (all from Gibco™ (Thermo Fisher Scientific Korea, Seoul, Republic of Korea)) at 37 °C and 5% CO_2_, with the medium replenished every 2 days. After serum starvation, 500 ng/mL of WT-bFGF and TS-bFGF diluted in DMEM containing 0.5% bovine calf serum (Gibco™ (Thermo Fisher Scientific Korea, Seoul, Republic of Korea)) were added to each well. After 72 h, 3-[4,5-dimethylthiazol-2-yl]-2,5-dephenyltetrazolium bromide (Sigma-Aldrich (Merck Korea, Seoul, Republic of Korea)) solution (5 mg/mL) was added to the wells, followed by incubation for 3 h. Di-methyl sulfoxide (Sigma-Aldrich (Merck Korea, Seoul, Republic of Korea)) was then added and absorbance was read at 570 nm. The values at each point were normalized by setting the values of control (fresh WT-bFGF or TS-bFGF not stored at RT) to 100%.

### 2.5. Cell Culture

HEK (human embryonic kidney) 293T cells (ATCC) were cultured in Dulbecco’s modified Eagle’s medium-high glucose (DMEM-high, Sigma-Aldrich) supplemented with 10% fetal bovine serum (FBS, Gibco™, Waltham, MA, USA) and 1% penicillin-streptomycin (P/S; Gibco™, Waltham, MA, USA). Cells were incubated at 37 °C in a humidified atmosphere containing 5% CO_2_. The hESCs line (SNUhES31) and OOM-SC-derived iPSC used in this study were approved by the Institutional Review Board (IRB) (7001355-201809-LR-267) of Konkuk University and conformed to the principles outlined in the Declaration of Helsinki. The hESC line was obatained from the Korean Centers for Disease Control and Prevention. Cells were maintained in Stemfit basic 03 (AJINOMOTO; Tokyo, Japan) medium containing either 20 ng/mL WT-bFGF (Peprotech, Cranbury, NJ, USA) or 20 ng/Ml TS-bFGF (in-house) with 1% MycoZap™ Plus-CL (Lonza, Cologne, Germany) (cell proliferation medium) on dishes coated with Matrigel Basement Membrane Matrix (Matrigel; Corning, Bedford, MA, USA). When cell confluency reached 70–80%, hESCs and iPSC colonies, the colonies were gently dissociated into single cells using Accutase (Stemcell Technologies, Seattle, WA, USA) at 37 °C for 5 min and then cultured in cell proliferation medium with 10 μM ROCK inhibitor, Y-27632 (MedChemExpress, Monmouth Junction, NJ, USA), on Matrigel-coated dishes. The medium was changed daily without Y-27632.

### 2.6. Luciferase Activity Assays Comparing WT-Bfgf and TS-Bfgf

To confirm the activity of WT-Bfgf and TS-Bfgf under culture conditions, luciferase activity in the HEK293 cells was measured using Additional Signaling Pathway Analysis (Promega, Madison, WI, USA) according to the manufacturer’s protocol. Heat-incubated Bfgf was adapted for 3 days under the same conditions as the cell culture conditions. In this study, the luciferase vector Pgl4.33[luc2p/SRE/Hygro] Vector was transfected using Polyjet™ in vitro DNA transfection reagent (SignaGen^®^ Laboratories, Frederick, MD, USA) according to the manufacturer’s instructions. The transfected cells were selected using 200 μg/mL hygromycin B (Thermo Fisher Scientific, Shanghai, China) for 24 h. The luciferase assay was performed using the Dual-Luciferase^®^ Reporter Assay System (Promega, USA) according to the manufacturer’s instructions. Luciferase activity was measured using SpectraMax iD3 (Molecular Devices, LLC, Taunton, MA, USA).

### 2.7. Cell Growth Kinetics

The hESC growth kinetics were determined as described in our previous study [24]. Briefly, cells were plated at 2 × 10^4^ cells/cm^2^, then seeded on Matrigel-coated 60 × 16 mm dishes (Thermo Fisher Scientific) along with cell proliferation medium. Cells were harvested after each passage (P), stained with 0.4% trypan blue solution (Gibco™, Roskilde, Denmark), and counted using a hemocytometer under a phase-contrast microscope. Cell population doubling was calculated according to the following equation: No (PD) = (log Nt − log N0)/0.301, where PD represents population doubling, Nt denotes the cell number after trypsinization/collection of cells, and N0 is the number of seeded cells.

### 2.8. RNA Isolation and Reverse Transcription-Quantitative Polymerase Chain Reaction

Total RNA was isolated from each group of hESCs using Labozol Reagent (Cosmo Genetech, Seoul, Republic of Korea) according to the manufacturer’s instructions, and purified RNA was quantified using a NanoDrop spectrophotometer (NanoPhotometer^®^ N50). cDNA synthesis was performed using 2 μg of total RNA with an M-MuLV reverse transcription kit (Cosmogentech, Seoul, Republic of Korea) and oligo-dT primers. The polymerase chain reaction (PCR) mixture was prepared using EzAmp™ qPCR 2X Master Mix (ELPIS-BIOTECH, Daejeon, Republic of Korea) and run on a Quant Studio™ 3 Real-Time PCR System (Thermo Fisher Scientific). The results were normalized using *GAPDH* expression as an internal control, and the fold change in gene expression was calculated using the comparative cycle time (Δct) method. The primers used in this study are listed in Table 1.

### 2.9. Cell Cycle Assay

To evaluate the cell cycle, hESCs were seeded at 5 × 10^5^ cells on a Matrigel-coated 60-mm dish and cultured for 24 h. Cells were harvested identically to the cell culture method, and a cycle analysis was performed using a NucleoCounter^®^ NC-250™ (Chemometec, Frederiksborg, Denmark) according to the manufacturer’s instructions. Cells were washed once with 1× PBS without Ca^2+^ and Mg^2+^ (Gibco™, USA), and the PBS was removed completely. The cells were gently pipetted using a lysis buffer (ChemoMetec) containing 10 μg/mL DAPI and incubated for 5 min in the same humidified atmosphere used for cell culture. Stabilization buffer (ChemoMetec, Denmark) was added to the lysed cells, loaded on NC-Slide A8™ (ChemoMetec, Denmark), and measured using NucleoView™ NC-250 software v1.11 (ChemoMetec, Denmark).

### 2.10. Alkaline Phosphatase (AP) Activity Test

AP staining was quantified using a Stemgent^®^ AP Staining Kit II (Stemgent™, Beltsville, MD, USA) following the manufacturer’s instructions. Briefly, hESCs were fixed with Fix solution at room temperature (RT) for 10 min and stained with an AP Staining Solution mixture (A, B, C, 1:1:1 ratio) at RT for 10 min in the dark. After two washes with 1× PBS without Ca^2+^ and Mg^2+^, the images of AP-positive colonies were evaluated microscopically (Leica Microsystems, Wetzlar, Germany; Dmi1).

### 2.11. Immunofluorescence Staining

For immunofluorescence staining, cells were fixed with 4% paraformaldehyde for 15 min at RT and then washed with 1× PBS. The cells were permeabilized with 0.5% Triton X-100 in 1× PBS without Ca^2+^ and Mg^2+^ for 10 min and blocked with 1× PBS with 1% bovine serum albumin (BSA; Bovogen, Australia) for 1 h at RT. The cells were incubated with specific primary antibodies, including anti-TRA-1-60 (1:200; Santa Cruz Biotechnology, Dallas, TX, USA, sc-21705) and anti-SSEA4 (1:200; Santa Cruz Biotechnology, sc-21704) at 4 °C overnight. The following day, the primary antibodies were removed and the cells were washed thrice with 1× PBS containing 0.1% Tween 20 for 10 min. Following this process, the cells were incubated with fluorescence-conjugated Alexa Fluor 488 (1:600; Invitrogen, Waltham, MA, USA, A-10680) secondary antibodies to detect the primary antibodies for 1 h at RT. Lastly, the cells were washed three times with 1× PBS containing 0.1% Tween20 and mounted using Vectashield with DAPI (Vector Laboratories, California). Fluorescent signals were observed using a confocal laser scanning microscope (Carl Zeiss, Göttingen, Germany, LSM900) and examined using Zen Blue Software 3.6. The fluorescence intensity was analyzed using ImageJ software v1.53t.

### 2.12. Spontaneous Differentiation

To achieve spontaneous differentiation, hESCs cultured in either WT-Bfgf or TS-Bfgf were harvested using the maintenance protocol and then seeded on AggreWell 400 (Stemcell Technologies, Vancouver, BC, Canada). The AggreWell plates were pretreated according to the manufacturer’s protocol with Anti-Adherence Rinsing Solution (Stemcell Technologies, Canada) and washed twice with 1× PBS without Ca^2+^ and Mg^2+^ (Gibco™, USA). The suspended cells were seeded at a density of 2 × 10^4^ cells/cm^2^ onto each well. After 24 h, embryonic bodies (EB) were gently transferred to a 100-mm petri dish (SPL Life Science, Republic of Korea) using a spontaneous differentiation medium (knockout DMEM/F12 (Gibco™, USA) + 20% FBS without bFGF). EBs were isolated on day 8 for RNA extraction. The medium was changed every two days. 

### 2.13. Hematopoietic Progenitor Cell (HPS) Differentiation

Differentiation of hESCs into HPS was carried out using an EB-based method, as previously described [24]. hESCs were seeded in a 96-well round-bottom plate (SPL Life Sciences, Republic of Korea) at a density of 1 × 10^4^ cells per well using APELL II (Stemcell Technologies, Canada) containing 40 ng/mL human Stem Cell Factor (SCF; Miltenyi Biotec, Cologne, Germany), 20 ng/mL human Vascular Endothelial Growth Factor (VEGF; R&D systems, Minneapolis, MN, USA), 20 ng/mL human Bone Morphogenetic Protein 4 (BMP-4; Miltenyi Biotec, Germany), and 10 μM Y-27632 for 11 days. The medium was replaced with fresh differentiation medium without Y-27632 and incubated for 18 h. 

### 2.14. Fluorescence-Activated Cell Sorting (FACS)

To compare the efficiency of differentiation into HPC, HPC-EB was dissociated with Accutase for 10 min at 37 °C in 5% CO_2_ incubator and then gently pipetted into single cells. After the dissociated cells were centrifuged for 5 min, the supernatant was aspirated and resuspended in the FACS buffer. The cells were then incubated with fluorescence-conjugated primary antibodies, anti-human CD34-FITC (1:100, Miltenyi Biotec, Germany), and anti-human CD45-APC (1:100, Miltenyi Biotec, Germany) for 30 min on ice. After three washes with FACS buffer, the fluorescence signal was measured using Beckman Coulter/CytoFLEX (Beckman Coulter, Indianapolis, IN, USA) and examined using CytExpert software (Beckman Coulter).

### 2.15. Generation of iPSCs from Mesenchymal Stem Cells (MSC)

For the generation of iPSCs, Orbicularis oculi muscle stem cells (OOM-SCs) were procured from Konkuk University Medical Center (KUMC 2019-05-043). OOM-SCs were cultured as sources of iPSCs. OOM-SCs were maintained following the method reported in our previous study [24]. Reprogramming factors (pCXLE-hOCT3/4-shp53, pCXLE-hSK, and pCXLE-hUL) were purchased from Addgene and transfected using the Neon Transfection System (Thermo Fisher Scientific, Waltham, MA, USA) following the manufacturer’s instructions. Each plasmid was delivered at a ratio of 1:1:1, for 3 μg in total. Transfected cells were seeded on pre-coated Matrigel (Corning, Bedford, MA, USA) plates and cultured in minimum essential medium (MEM)-α (Gibco™, USA) supplemented with 10% FBS (Gibco™, Waltham, MA, USA) and 1% P/S (Gibco™, Waltham, MA, USA) for three days. After three days, the medium was completely replaced with a hESC proliferation medium containing WT-bFGF (Peprotech, USA) or TS-bFGF with 10 µM Y-27632 (MedChemExpress, USA). The medium was replaced every 2 days until iPSC colony formation. The colonies were transferred into four-well plates (SPL Life Sciences, Pochon, Republic of Korea) coated with Matrigel (Corning, Bedford, MA, USA), then cultured according to the hESC culture method.

### 2.16. Statistical Analysis

All statistical analyses were performed using GraphPad Prism v7.0 (GraphPad Software). In most experiments designed in triplicate, adjusted *p*-values were calculated through Student’s *t*-test, one-way ANOVA, and two-way ANOVA using GraphPad Prism. In all figures, the *p*-values are marked with asterisks (* *p* < 0.05, ** *p* < 0.01, *** *p* < 0.001, **** *p* < 0.0001).

## 3. Results

### 3.1. Schematic Diagram of Growth Factor Construction and Comparative Evaluation of Activity

**Figure 1:**
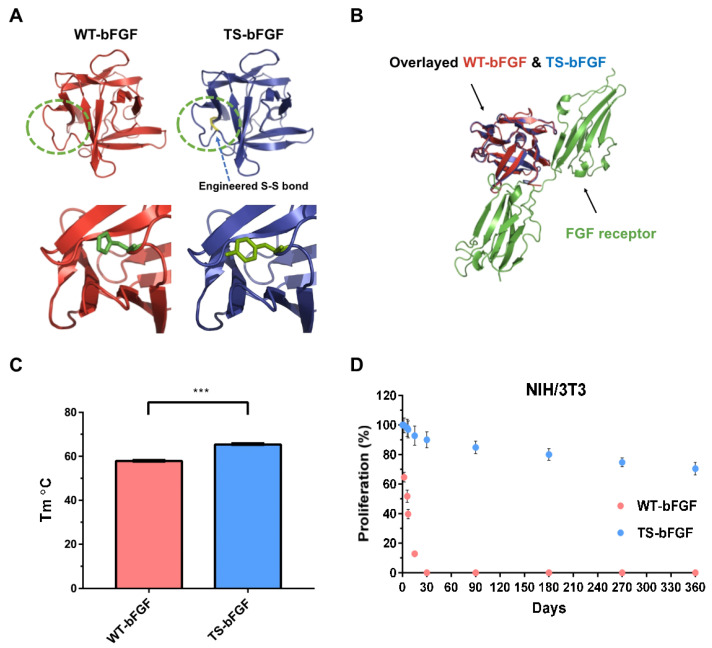
Computational design of thermostable-bFGF (TS-bFGF) and its comparison with wild-type bFGF (WT-bFGF) in terms of stability. (**A**) Molecular images of WT-bFGF and TS-bFGF carrying an engineered disulfide (S-S) bond. The dotted green circles indicate the regions engineered in detail. (**B**) Docking images of WT-bFGF and TS-bFGF with the FGF receptor. (**C**) Thermal stability (Tm, melting temperature) of WT-bFGF and TS-bFGF. (**D**) Cell proliferation activity of WT-bFGF and TS-bFGF in NIH/3T3 cells stored in solution at 25 °C for a period of 12 months. The values at each point were normalized by setting the values of control (a fresh WT-bFGF of TS-bFGF, not stored at 25 °C) as 100%. *** *p* < 0.001.

**Figure 2:**
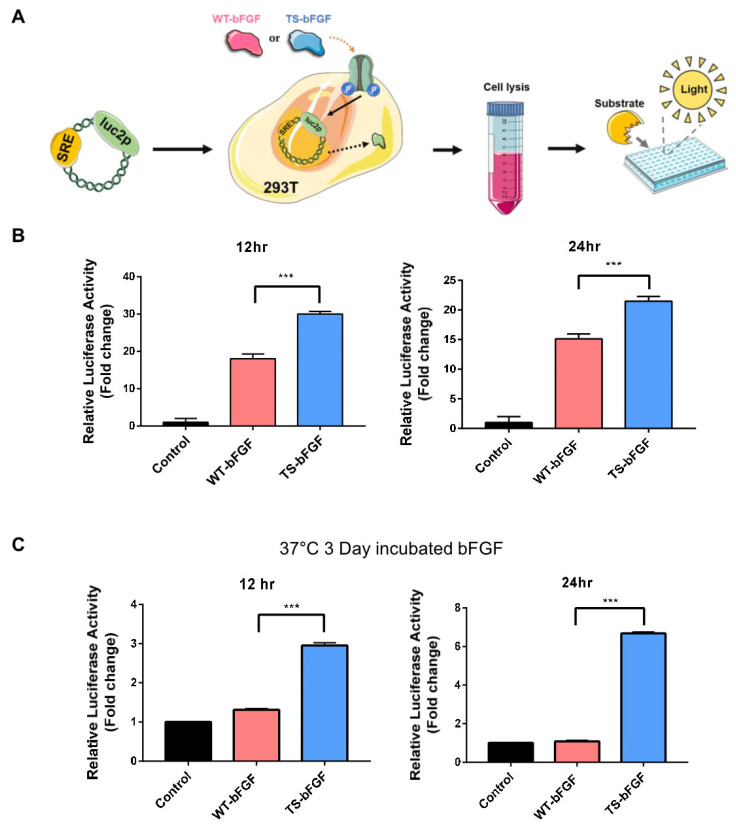
Comparison of luciferase activity under culture conditions in 293T cells. (**A**) Schematic diagram of the luciferase reporter gene constructs and scheme (SRE with luc2p and serum-response element with luciferase gene, respectively. (**B**) Luciferase activity of reporters carrying either WT-bFGF or TS-bFGF were measured in hESC. (**C**) Luciferase activity of reporters carrying either the heat-incubated WT-bFGF or TS-bFGF were measured in hESCs at 12 and 24 h. Values are expressed as the mean ± SEM of three independent experiments. *** *p* < 0.001.

Structure-based computational techniques provide a powerful tool in designing engineered proteins with improved function or stability. To accomplish these purposes, the free energy (ΔG, kcal/mol) of the system should be calculated correctly and verified experimentally. Alteration of amino acids results in changes of ΔG (ΔΔG) that are concomitant with enhanced or decreased thermostability, with negative values of ΔΔG indicating stabilization. We selected fifteen mutations and calculated their ΔΔG values using FoldX (see Supplementary Figure S1). Mutations with high negative ΔΔG values were selected, and the respective stabilities of the mutants were experimentally analyzed by T_m_ measurement. The experimentally confirmed stabilizing mutations were selected and combined to find the finally-selected mutant (TS-bFGF) (Figure 1A). The TS-bFGF contains one disulfide bond and four-point mutations (A1G/C77S/C95S/A83C/H58Y), with a ΔΔG value of −4.384; the C77S and C95S mutations were added to inhibit inter-molecular aggregation, and the A1G mutation was added to optimize the production process. Docking models of TS-bFGF or WT-bFGF with the FGF receptor generated through molecular modeling tools (Z dock and amber) revealed that the two bFGFs closely overlapped and fit well into the binding pocket of the FGF receptor (Figure 1B). The superiority of TS-bFGF over WT-bFGF was clearly demonstrated by the enhanced melting temperature (T_m_), from 58 °C to 65 °C (Figure 1C), and the maintenance of the activity in solution at room temperature (25 °C) for a long term (up to 12 months), which is an important characteristic that is lacking in WT-bFGF (Figure 1D). To further investigate whether TS-bFGF could have more intracellular effects than WT-bFGF through the FGF receptor, a luciferase activity assay was performed. The luciferase assay is an experimental method that can quantitatively measure the effect of genetic mutations on gene expression. Dual-luciferase assays have been widely used to assess the transcriptional activities of transcriptional regulatory sequences and the factors that regulate them (Figure 2A). Various bFGFs were tested in HEK293 cells transfected with a luciferase expression vector. TS-bFGF was more active than WT-bFGF in a time-dependent manner (Figure 2B). The promoter in HEK293 cells operated more effectively with TS-bFGF than with WT-bFGF. In particular, it was confirmed that TS-bFGF was stably and time-dependently activated even after heat incubation (Figure 2C).

### 3.2. TS-bFGF Increases Embryonic Stem Cell Growth Kinetics

**Figure 3:**
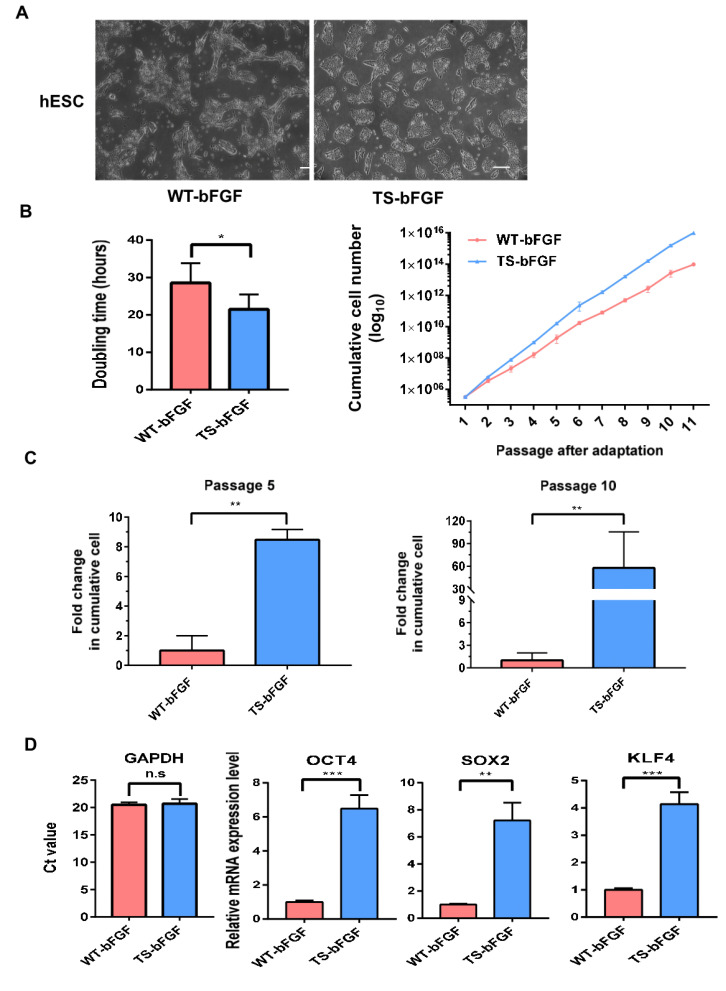
Analysis of the comparative effect of hESC proliferation and stemness using WT-bFGF and TS-bFGF. (**A**) Phase contrast images of hESC colonies grown under different bFGF conditions on feeder-free layers on day 2. (**B**) Comparative analysis of growth rates of hESCs treated with WT-bFGF and TS-bFGF in continuous cell culture. (**C**) Comparison of cumulative population cell number over time in WT-bFGF-cultured and TS-bFGF-cultured hESCs in short-term (passage 5) or long-term (passage 10) cell culture. (**D**) Relative quantification of OCT4, SOX2, and KLF4 mRNA levels of the hESC colonies on day 3 of culture with WT-bFGF or TS-bFGF. Scale bar: 200 µm. Values are expressed as the mean ± SEM of three independent experiments. n.s: not significant, * *p* < 0.05, ** *p* < 0.01, *** *p* < 0.001.

hESCs are the most potent candidates for the replacement of damaged cells and/or tissues in the target organ, although obstacles such as the assurance of guided differentiation and control of the immune response following cell therapy continue to exist in the clinical application of hESCs. The stable maintenance of hESCs is essential for the development of appropriate culture conditions using beneficial growth factors. We analyzed whether hESCs were stably maintained when using TS-bFGF in comparison with WT-bFGF. TS-bFGF-treated hESCs were able to form undifferentiated colonies at a higher rate than the control group (Figure 3A). It was confirmed that the cell doubling time was improved in the hESC group treated with TS-bFGF compared to the control group, and it was shown that TS-bFGF affected the proliferation of hESCs through long-term culture (Figure 3B). In addition, when analyzing the effect on the number of hESCs in short- or long-term culture after adding TS-bFGF to the cell culture medium, it was confirmed that the cumulative number of hESCs increased in both the short-term (8-fold change) and long-term (57-fold change). Accordingly, we confirmed that TS-bFGF had a positive effect on hESC proliferation (Figure 3C). To be effective, hESCs must maintain an undifferentiated morphology and retain their pluripotency [25]. According to the results of many studies, hESCs are able to differentiate into various functional cells, and their potential as a treatment for various diseases has been reported as well [26,27,28]. Therefore, it is important to maintain the line functionality of hESCs. To this end, we compared the maintenance of long-lasting stemness in hESC cultures using WT-bFGF and TS-bFGF. When hESCs were cultured using TS-bFGF, it was confirmed that the stem cell properties of the stemness markers OCT4, SOX2, and KLF4 were four to six times higher than those of the control group (Figure 3D). 

### 3.3. Effect of TS-bFGF on hESC Stemness Function

**Figure 4:**
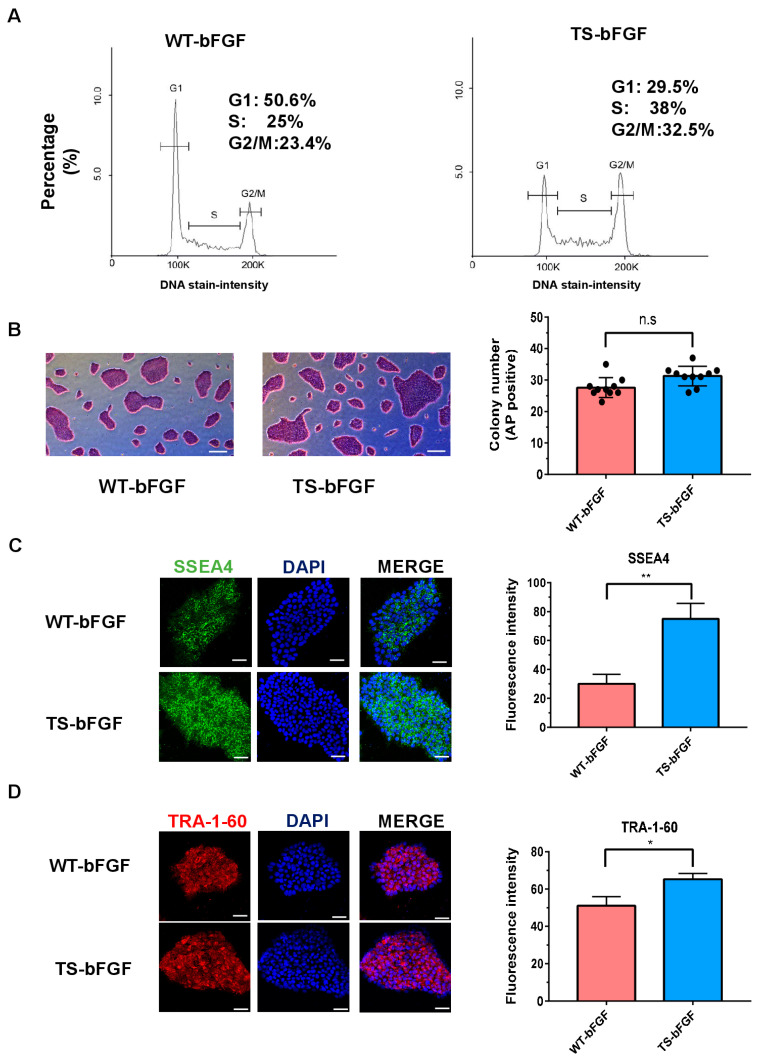
Analysis of main characteristics of hESCs cultured with TS-bFGF. (**A**) Effects of WT-bFGF and TS-bFGF on hESC cycle. (**B**) hESC colonies were positively stained with alkaline phosphatase. Immunofluorescence staining was performed to detect pluripotent markers (Scale bar: 200 μm). (**C**,**D**) Representative immunofluorescence images showing the expression of pluripotency markers (SSEA4 and TRA-1-60) in hESCs cultured with either TS-bFGF or WT-bFGF. Nuclei were stained with DAPI (Scale bar: 50 μm). Values are expressed as the mean ± SEM of three independent experiments. n.s: not significant, * *p* < 0.05, ** *p* < 0.01.

As shown in Figure 3, cell proliferation increased when hESCs were cultured with TS-bFGF. Considering that maintaining the cell cycle and pluripotent state is a focus of ongoing research into hESCs [29], we evaluated and compared the effects of TS-bFGF and WT-bFGF on hESC stemness and cell cycle. When comparing the effects of growth factors on the cell cycle, it was confirmed that the S phase (intracellular DNA replication) and G2-M phase (mitosis) were further activated in hESCs cultured with TS-bFGF (Figure 4A), suggesting that TS-bFGF positively affects cells related to the proliferation of hESCs. When comparing the effects of TS-bFGF and WT-bFGF on the stemness of hESCs by using alkaline phosphatase (AP) staining as part of the stemness evaluation method, similar results were obtained (Figure 4B). This result demonstrates that TS-bFGF acts similarly to the existing WT-bFGF even though hESCs maintain their stemness. SSEA4 and TRA-1-60 are defined as embryonic stem cell hallmarks of pluripotency. They have been shown to be unique biomarkers of pluripotent stem cells, ceasing to be expressed upon differentiation [30,31]. Expression of the pluripotency markers SSEA4 and TRA1-60 confirmed that hESCs were more effective in maintaining pluripotency under TS-bFGF culture compared to WT-bFGF (Figure 4C,D). Considering these results together, we can confirm that TS-bFGF is superior to WT-bFGF in maintaining pluripotency and function of cultured hESCs.

### 3.4. High Rate of Spontaneous Trilineage Differentiation Potential in TS-bFGF-Treated Embryoid Bodies

**Figure 5:**
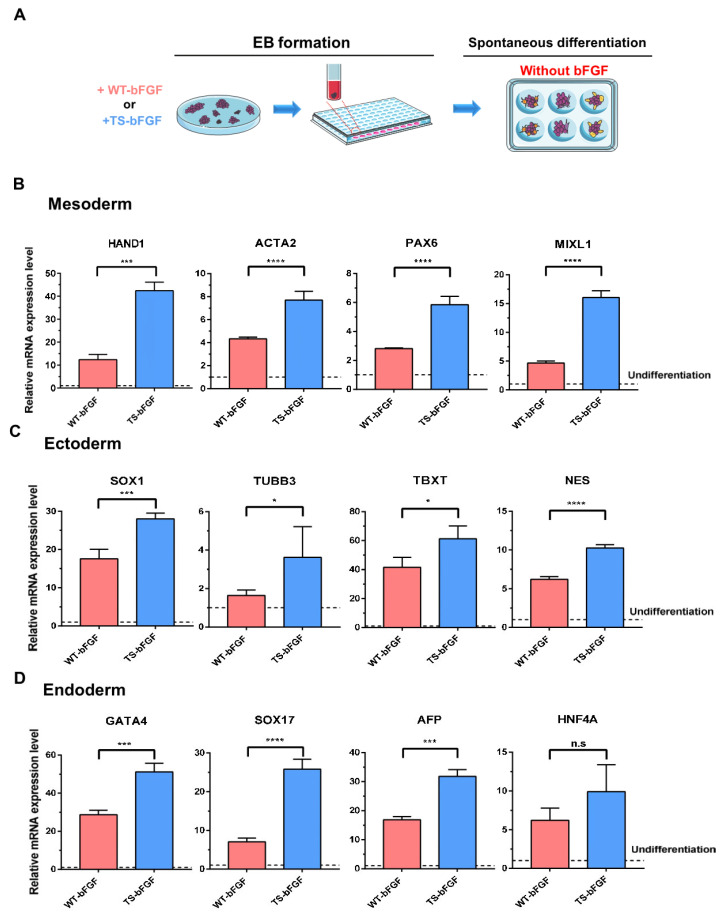
Three-lineage differentiation efficiency of hESCs as indicated by gene expression. (**A**) Schematic of the EB formation procedure. One day before initiation of differentiation, WT-bFGF or TS-bFGF was added to the hESCs, which were then transferred into AggreWell plates at 24 h. The EBs formed through spontaneous differentiation were transferred into untreated 6-well plates without bFGF. Comparative evaluation of enhanced differentiation capacity into mesoderm (**B**), ectoderm (**C**), and endoderm (**D**) of WT-bFGF or TS-bFGF-treated hESC-derived EBs. Values are expressed as the mean ± SEM of three independent experiments. n.s: not significant, * *p* < 0.05, *** *p* < 0.001, **** *p* < 0.0001.

An essential alternative to pluripotency is an in vitro approach involving the generation of embryoid bodies (EBs) from hESCs [32]. Differentiation of pluripotent stem cells into various functional cells after hESC formation in EBs is of great significance. As EB formation is an essential step in many embryonic stem cell (ESC) differentiation methods, the reported microwell and suspension culture methods were used in this study [33]. WT-bFGF and TS-bFGF were added during hESC culture and the methods used for spontaneous trilineage differentiation after EB formation were described (Figure 5A). We evaluated the effects of TS-bFGF and WT-bFGF on differentiation into mesoderm, endoderm, and ectoderm during spontaneous differentiation following EB formation. To determine the status of cells differentiated from hESCs, we assayed the expression of markers of lineage-specific cell differentiation. During mesoderm differentiation from EBs generated using TS-bFGF, the level of expression of the genes HAND1, ACTA2, TBXT, and MIXL1 was two to twenty times higher than in the WT-bFGF group (Figure 5B). In addition, the ectoderm-specific gene markers SOX1, TUBB3, PAX6, and NES were more highly expressed in hESCs cultured using TS-bFGF (Figure 5C). A similar effect was found in the endoderm-specific gene markers GATA4, SOX17, AFP, and HNF4A (Figure 5D). These results confirm that TS-bFGF consistently shows positive effects on the mesoderm, endoderm, and ectoderm during spontaneous trilineage differentiation using EBs formed from hESCs. 

### 3.5. TS-bFGF Improved Differentiation of hESCs to Hematopoietic Progenitor Cells (HPCs)

**Figure 6:**
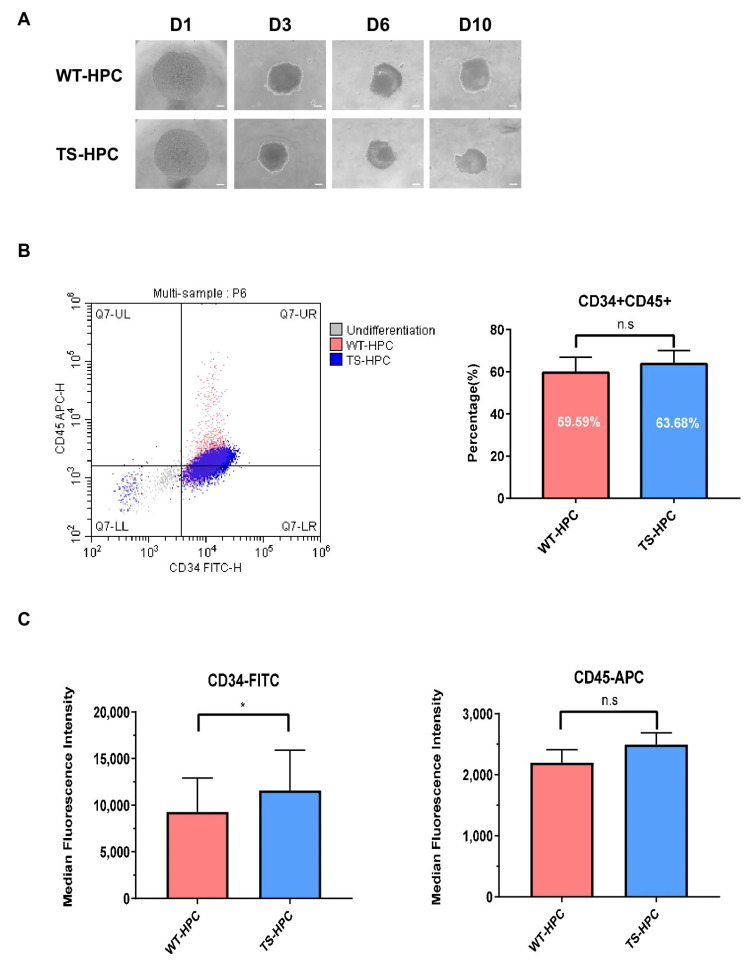
Comparative analysis of WT-bFGF and TS-bFGF in terms of the differentiation of hESCs into HPCs. (**A**) Representative images of differentiated HPCs from day 0 to 10 (scale bar: 100 µm). (**B**) The CD34+ and CD45+ populations were identified through flow cytometry of HPC-EBs. (**C**) The fluorescence expression intensity of CD34 and CD45, respectively. n.s: not significant, * *p* < 0.05.

In an appropriate culture system, embryonic stem cells can self-renew and differentiate into hematopoietic lineages, including hematopoietic stem cells (HSCs), hematopoietic progenitor cells (HPCs), and mature hematopoietic cells [34]. Verification of hematopoietic cells typically involves the use of a cell surface marker such as CD34 to identify putative HPC populations within the mixed-cell population [35]. The effect of TS-bFGF on differentiation into HPC (TS-HPC) was compared with that of WT-bFGF (WT-HPC). During HPC differentiation using TS-bFGF and WT-bFGF, the external changes of the cells were confirmed daily under a microscope. On the tenth day of differentiation, HPC-EB was found to be maintained, and there were no morphological differences between the WT-bFGF and TS-bFGF treatment groups. (Figure 6A). To compare the differentiation efficiency, the differentiation rate of each HPC-EB was confirmed through cell surface analysis using the HPC surface markers CD34 and CD45. When analyzing cells expressing both CD34+ and CD45+, a slight increase was confirmed in the group treated with TS-bFGF (Figure 6B). In particular, by analyzing the expression intensity of CD34 (a representative marker of HPC), it was found that the TS-bFGF-cultured hESCs exhibited approximately 30% higher fluorescence intensity than the WT-bFGF group (Figure 6C), suggesting that TS-bFGF can affect the differentiation rate to a greater degree than WT-bFGF during the differentiation of hESCs into HPCs.

### 3.6. Effects of TS-bFGF on the Proliferation and Stemness of Induced Pluripotent Stem Cells (iPSCs)

**Figure 7:**
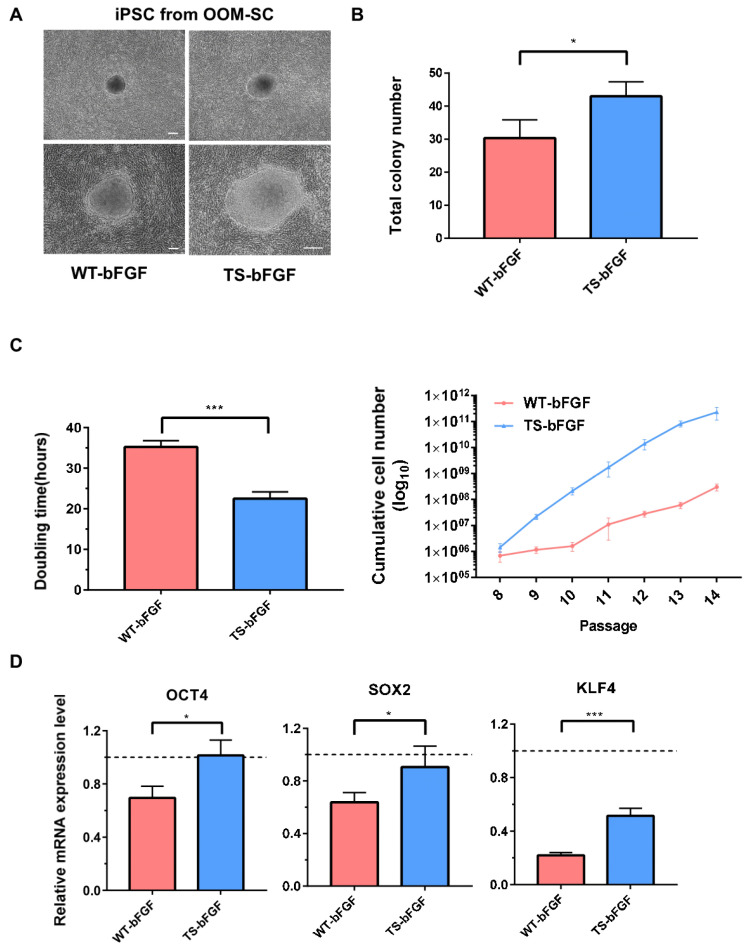
Confirmation of efficacy through comparison of iPSC generation, maintenance, and stemness. (**A**) Generated iPSC colony morphology and (**B**) total number of colonies according to each bFGF from OOM-SCs. (**C**) Comparative analysis of growth rate of iPSCs treated with WT-bFGF or TS-bFGF in cell culture. (**D**) Evaluation of stemness gene expression levels in iPSC at passage 10, using hESCs treated with WT-bFGF as control. Scale bar: 200 µm. Values are expressed as the mean ± SEM of three independent experiments. * *p* < 0.05, *** *p* < 0.001.

Induced pluripotent stem cells (iPSCs) have emerged as a powerful tool in regenerative medicine thanks to their ability to differentiate into a wide range of cell types [36], which is invaluable for developing patient-specific therapies. In this context, maintaining a stable cell culture is crucial. After confirming the efficacy of TS-bFGF in ESCs, we analyzed its effect on iPSCs in comparison to WT-bFGF. When generating iPSCs with TS-bFGF, we observed a distinct change in the outer appearance of the cells (Figure 7A) and total colony number (Figure 7B) compared to the control group. Furthermore, the passage time of cells was reduced by more than threefold, and the accumulated cell proliferation increased by over 100-fold with repeated passages, demonstrating a significant difference (Figure 7C). Through the use of TS-bFGF for iPSC culture, we were able to maintain sustained stem cell function. As a result, we observed a significant increase in the mRNA levels of the stem cell markers OCT4, SOX2, and KLF4, which are commonly used for evaluating stem cell function in comparison to the control group (Figure 7D). These results suggest that an improved culture environment can be created when culturing pluripotent stem cells using TS-bFGF, leading to enhanced cell performance.

## 4. Discussion

Due to the critical role of bFGF in the maturation of hESCs in an undifferentiated state, there is currently, great interest in improving its inherently low thermal stability, and considerable efforts are being made towards this end. The low thermal stability of bFGF leads to rapid loss of activity during hESC culture, limiting the application of bFGF in large-scale cell cultures used to produce stem cell-based products. Among the many possible strategies for stabilizing bFGF [37], we have adopted a chemical modification approach, resulting in the successful creation of a novel thermostable bFGF variant, TS-bFGF, by introducing point mutations into specific positions predicted by computer-assisted structural modeling. Previously, we have demonstrated that due to the increased thermal stability of TS-bFGF, its structure is able to remain unchanged for up to 12 months in solution at room temperature, resulting in better efficacy in wound healing [18]. In this study, we have further shown that TS-bFGF is superior to WT-bFGF in term of maintaining the cell proliferation activity in solution at room temperature over the long term, with the activity of TS-bFGF remaining almost fully intact even after a year (~85%), while in WT-bFGF little activity remained after only a month. These results reveal that the better efficacy in wound healing of TS-bFGF observed previously is likely due to the prolonged maintenance of cell proliferation activity in TS-bFGF. bFGF variants with increased thermal stability have been developed through a similar approach, and these bFGF variants have shown improved outcomes in terms of proliferation and differentiation [38,39]. However, most of these bFGF variants were created by substituting amino acid residues in a large number of positions (as much as or more than 10% of the sequence); in contrast, TS-bFGF was engineered to carry a disulfide bond that is structurally buried inside and contains only four mutations, dissimilar from WT-bFGF, while providing similar culture outcomes. It is generally agreed that the introducing a greater number of mutations, results in a higher mutational burden, potentially leading to various problems in bioprocesses, such as difficulty in formulation, low production yield, and a higher risk of immunogenicity when administered as a therapeutic. Considering the drawbacks of thermostable bFGF variants with high mutation loads, TS-bFGF, which is shown here to be thermostable and effective in the differentiation process, represents a preferred option.

In our luciferase-based cell proliferation assays, TS-bFGF showed slightly higher activity compared to WT-bFGF, and this was even more dramatic when the two bFGFs were tested after incubation at 37 °C for 3 days, during which time the efficacy of WT-bFGF was completely diminished (likely because of its lower stability). As seen in the docking models of the two bFGFs with the FGF receptor, they closely overlap with each other and fit into the well of the receptor-binding pocket. Therefore, the reason for TS-bFGF showing a higher activity compared to WT-bFGF is more likely due to the improved thermostability of TS-bFGF than to a tighter fit of TS-bFGF with the FGF receptor, though we cannot exclude this possibility. It is reasonable to assume that WT-bFGF loses its activity gradually during the assay, whereas TS-bFGF maintains its activity over time, which is supported by the assay comparing the two bFGFs after incubation at 37 °C for 3 days, during which time the activity of WT-bFGF was completely lost. However, the link between the thermal stability of TS-bFGF and FGF signaling is not yet clear, and further investigation is required to answer this question. We believe that the same reasoning can be applied to explain the higher rate of cell division, resulting in a greater number of accumulated cells and higher levels of stemness markers in the TS-bFGF-treated hESCs compared with the WT-bFGF-treated controls. More interestingly, we observed that hESCs cultured with TS-bFGF showed higher levels of trilineage differentiation markers during spontaneous differentiation compared to the controls treated with WT-bFGF. It is not yet clear whether this could be due to the healthier and younger status of the hESCs cultured with TS-bFGF compared to the WT-bFGF-treated control; this might make hESCs treated with TS-bFGF able to differentiate more actively by expressing the differentiation markers. It would be interesting to investigate how FGF signaling is regulated to initiate differentiation after removal of TS-bFGF, which might reveal the difference between the two bFGFs in terms of FGF signaling during cellular differentiation.

The potential of hESCs as targeted therapy to treat various diseases, including dystrophic epidermolysis bullosa, spinal muscular atrophy, and Huntington’s disease, has been demonstrated in a number of studies [21,28,33]. hESCs can differentiate into somatic cells, and can be used as a replacement for damaged cells [27]. In addition, the genetic mechanisms of diseases, in vitro disease models, and test drugs can be explored using hESCs [40]. Various protocols have been developed to differentiate hESCs into target cells in vitro [4]. Culturing hESCs at a higher yield while maintaining their stemness is essential for differentiation into desired cells. Therefore, based on the results of our study, it is expected that TS-bFGF can play an important role in the stem cell industry and biomedical fields, where high-quality stem cells are essential. 

Although hESCs represent a valuable tool, yielding desirable cells that can be applied in many biotechnological fields, further development of hESCs has been impeded due to ethical issues [41,42]. To overcome this problem, biotechnological fields that utilize induced pluripotent stem cells (iPSCs), which are derived from differentiated cells, have been growing rapidly, and continue to expand [43]. Because iPSCs are free from the ethical issues associated with hESCs, and are essentially the same as ESCs in terms of their ability to differentiate into desired cells, the potential of iPSCs is increasing. In this study, we have demonstrated that TS-bFGF can be applied to iPSCs in the same manner as hESCs; iPSCs (derived from hOOM-SCs) cultured with TS-bFGF could give rise to faster cell division, resulting in higher accumulated cell numbers and higher levels of stemness markers (OCT4, SOX2, and KLF4). Considering the overall growth potential of iPSCs, it is doubtless that the use of TS-bFGF in the stem cell industry will increase as well.

## 5. Conclusions

Taken together, the results of our study show that TS-bFGF, a thermostable and long-acting bFGF, improves the culture outcomes of human pluripotent stem cells (both hESCs and iPSCs) in terms of cell proliferation, stemness, and differentiation. Considering the growth potential of high-quality pluripotent stem cells in biomedical applications, we believe that the use of TS-bFGF in the stem cell industry is likely to increase as well. 

## 6. Patents

We filed patents with regard to this study (Korean patent application number 10-2023-0069536; Korean patent application number 10-2023-0071437).

## Figures and Tables

**Table 1:** Primer information.

Gene	Primer Sequence ^1^ (5′ → 3′)	Product Size (bp)	Accession No.
GAPDH	GTCTCCTCTGACTTCAACAGCG (F) ACCACCCTGTTGCTGTAGCCAA (R)	131	NM_001357943.2
OCT4	CCTGAAGCAGAAGAGGATCACC (F) AAAGCGGCAGATGGTCGTTTGG(R)	106	NM_203289.6
SOX2	GCTACAGCATGATGCAGGACCA (F) TCTGCGAGCTGGTCATGGAGTT (R)	135	NM_003106.4
KLF4	CATCTCAAGGCACACCTGCGAA (F) TCGGTCGCATTTTTGGCACTGG (R)	156	NM_001314052.2
HAND1	CAAGGATGCACAGTCTGGCGAT (F) GCAGGAGGAAAACCTTCGTGCT (R)	117	NM_004821.3
ACTA2	CTATGCCTCTGGACGCACAACT (F) CAGATCCAGACGCATGATGGCA (R)	115	NM_001406462.1
PAX6	CTGAGGAATCAGAGAAGACAGGC (F) ATGGAGCCAGATGTGAAGGAGG (R)	131	NM_000280.6
MIXL1	CCCGACATCCACTTGCGCGAG (F) GGAAGGATTTCCCACTCTGACG (R)	118	NM_031944.3
SOX1	GAGTGGAAGGTCATGTCCGAGG (F) CCTTCTTGAGCAGCGTCTTGGT (R)	136	NM_005986.3
TUBB3	TCAGCGTCTACTACAACGAGGC (F) GCCTGAAGAGATGTCCAAAGGC (R)	120	NM_001197181.2
TBXT	CCTTCAGCAAAGTCAAGCTCACC(F) TGAACTGGGTCTCAGGGAAGCA (R)	153	NM_047419269.1
NES	TCAAGATGTCCCTCAGCCTGGA (F) AAGCTGAGGGAAGTCTTGGAGC (R)	106	NM_006617.2
GATA4	GCGGTGCTTCCAGCAACTCCA (F) GACATCGCACTGACTGAGAACG (R)	139	NM_001308093.3
SOX17	ACGCTTTCATGGTGTGGGCTAAG (F) GTCAGCGCCTTCCACGACTTG (R)	112	NM_022454.4
AFP	GCAGAGGAGATGTGCTGGATTG (F) CGTGGTCAGTTTGCAGCATTCTG (R)	113	NM_001354717.2
HNF4A	GGTGTCCATACGCATCCTTGAC (F) AGCCGCTTGATCTTCCCTGGAT (R)	144	NM_000457.6

^1^ F and R indicate forward and reverse primers, respectively.

## Data Availability

Not applicable.

## Supplementary Materials

The following supporting information can be downloaded at: https://www.mdpi.com/article/10.3390/biology12060888/s1, Supplementary Figure S1: 15 selected mutation sites and the corresponding ΔΔG values calculated using FoldX.
