# Supplementary material for: Thermostable Human Basic Fibroblast Growth Factor (TS-bFGF) Engineered with a Disulfide Bond Demonstrates Superior Culture Outcomes in Human Pluripotent Stem Cell
Source: Biology (Basel). 2023 Jun 20;12(6):888. doi: 10.3390/biology12060888 (PMC10294964; doi:10.3390/biology12060888)
Supplement: Supplementary file 1 [file biology-12-00888-s001.zip › biology-2407791-supplementary.pdf]

Supplementary Figure S1

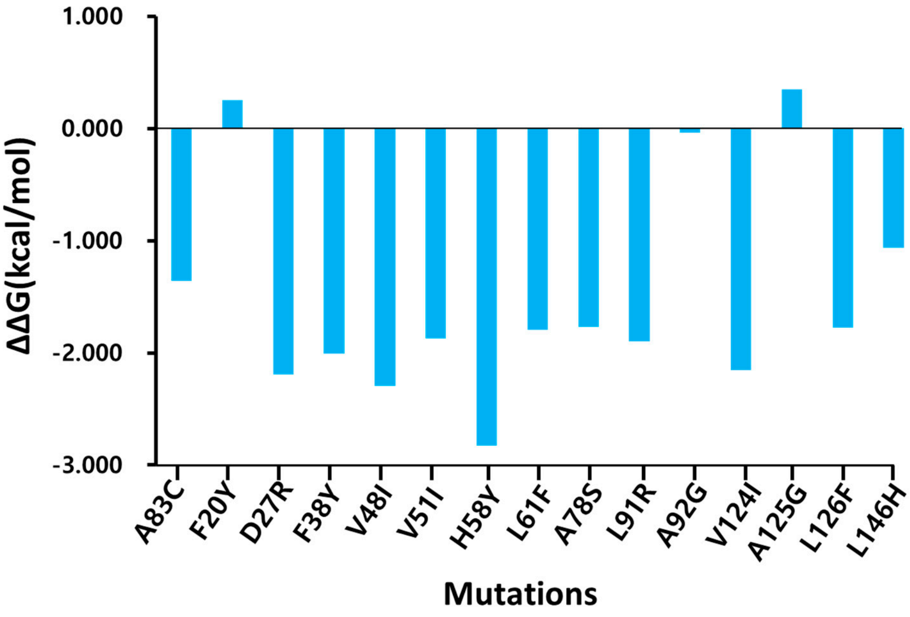

Supplementary Figure S1. 15 selected mutation sites and the corresponding ΔΔG values calculated using FoldX.
